# How Does AI Affect College? The Impact of AI Usage in College Teaching on Students’ Innovative Behavior and Well-Being

**DOI:** 10.3390/bs14121223

**Published:** 2024-12-19

**Authors:** Ke Ma, Yan Zhang, Beihe Hui

**Affiliations:** 1 Sports Teaching and Research Department, Zhejiang Sci-Tech University, Hangzhou 310018, China; make028@zstu.edu.cn; 2 College of Sports, Nanjing Tech University, Nanjing 211816, China; 13655166375@163.com

**Keywords:** the usage of AI in teaching, positive emotion, innovative behavior, well-being, AI trust

## Abstract

Currently, there is a growing trend for college and university teachers to use AI in their teaching work. However, existing research explores the impact of teachers’ usage of AI in the workplace on students. Based on resource preservation theory, this study examined the mechanism of the usage of AI in teaching on students’ innovative behavior and well-being with a sample of 356 college students from Zhejiang Province. The study found that the usage of AI in teaching significantly and positively affected students’ innovative behavior and well-being, with students’ positive emotion playing a mediating role. Students’ AI trust not only moderated the effect of the usage of AI in teaching on positive emotion, but also moderated the mediating role of positive emotion. The findings have important implications for teachers’ instructional management and practice.

## 1. Introduction

In the context of highly developed information technology today, artificial intelligence (AI) has emerged as an innovative educational tool, progressively becoming a crucial support in higher education [1]. The utilization of AI by educators to enhance teaching methods and resource management is no longer merely a novel experiment but a pressing necessity [2]. As global efforts continue to pursue enhanced educational quality, the application of AI technologies not only efficiently improves teaching effectiveness but also promises to inspire new insights and opportunities for student learning experiences [3].

Against this backdrop, an increasing amount of research has begun to focus on how teachers employ AI in teaching and its impact on students [4]. The existing literature indicates that teachers’ use of AI not only significantly enhances students’ academic performance but also potentially profoundly influences their daily behaviors and states. However, most studies focus on regions with specific sociocultural characteristics, limiting the generalizability of findings. To address this, this study includes discussions of research from diverse geographic and sociocultural contexts to explore the transferability of AI’s impact in education across varied environments [5,6,7]. Innovative behavior and well-being are critical student outcome indicators that not only relate to individuals’ learning experiences and sense of achievement but also directly contribute to comprehensive improvements in educational quality [8,9,10]. However, current research overlooks the exploration of how teachers’ usage of AI in teaching affects students’ innovative behavior and well-being. To address this research gap, this study aims to investigate the impact of teachers’ usage of AI in teaching on students’ innovative behavior and well-being.

Conservation of resources theory provides a robust theoretical framework for understanding the mechanisms through which teachers’ usage of AI in teaching influences students. Additionally, pedagogical theories, such as transformative learning and constructivist education, highlight that education involves not only cognitive and emotional dimensions but also ethical, critical, and sociocultural aspects. Integrating these perspectives ensures a more holistic understanding of AI’s role in education [11]. According to this theory, individuals adapt to environmental changes by adjusting their use of internal resources, thereby effecting their behaviors and psychological states [11]. In educational settings, positive emotion serves as a crucial internal resource that not only directly influences students’ emotional experiences but may also act as a mediator during AI interventions, further modulating and amplifying its effects on learning outcomes [11,12,13]. Therefore, this study intends to explore the mediating role of positive emotion in the process by which teachers’ usage of AI in teaching influences students’ innovative behavior and well-being.

Additionally, the degree of trust individuals place in AI is viewed as another important moderating factor. In educational environments, students’ trust in teachers’ use of AI not only affects their acceptance of AI technologies but also moderates the positive effects they derive through positive emotion [6,14,15]. Hence, understanding and exploring the role of AI trust in the teaching process is crucial for fully leveraging the potential of AI in education, holding significant theoretical and practical implications [16,17,18]. Consequently, this study aims to investigate the boundary conditions of AI trust in the process by which teachers’ usage of AI in teaching influences students’ innovative behavior and well-being.

In conclusion, this study aims to delve into the specific mechanisms through which teachers’ usage of AI in teaching affects students’ innovative behavior and well-being, based on resource conservation theory. It seeks to examine the mediating role of positive emotion and the moderating role of AI trust. Building on this foundation, the study further extends the application of resource conservation theory in educational contexts and provides new theoretical support and practical insights for teachers’ instructional management and practice.

## 2. Theoretical Foundations and Hypothesis Development

### 2.1. Theoretical Foundations

The conservation of resources theory is a theory in cognitive psychology that primarily investigates how individuals balance the use of cognitive resources when processing information [11]. This theory posits that individuals strive to minimize the expenditure of cognitive resources during cognitive tasks to ensure efficient utilization of resources in complex tasks. Specifically, the conservation of resources theory emphasizes several key points. First, the limited nature of cognitive resources. This theory asserts that human cognitive systems are finite and cannot indefinitely sustain the burden of information processing. Second, task load and resource allocation. According to this theory, individuals allocate cognitive resources based on the difficulty and complexity of tasks when handling multiple or complex tasks. Third, resource conservation and efficiency. The theory suggests that individuals tend to conserve cognitive resources by enhancing cognitive efficiency and reducing redundant information. Fourth, task priority and resource allocation. The theory proposes that individuals allocate cognitive resources based on the priority of tasks, prioritizing the processing of important tasks [7]. The conservation of resources theory plays a significant role in understanding resource allocation, decision-making, and execution processes in cognitive processes, particularly in explaining how individuals exercise cognitive control and make decisions in complex situations.

### 2.2. The Usage of AI in Teaching and Students’ Innovative Behavior/Well-Being

The usage of AI in teaching can help reduce some routine and repetitive tasks, such as homework correction, classroom management, etc., thus releasing more cognitive resources that can be used to promote the development of students’ innovative thinking and creativity [19]. Such released cognitive resources can allow students to focus more on understanding, application, and innovation. AI technology can help teachers identify and respond to students’ learning needs more effectively through features such as personalized learning, intelligent assistance, and feedback [20]. This personalized and precise support can make students more confident and efficient in the learning process, thus enhancing their motivation and innovation [21]. In addition, teachers using AI technology can better manage teaching resources and time allocation, and devote more time to heuristic teaching, exploratory learning, and guidance in innovative projects. Such optimized task allocation and resource management can help develop students’ ability to solve problems, discover new knowledge, and innovate [22]. Finally, when teachers are able to provide a more personalized, challenging, and inspiring learning experience through AI technology, students’ learning outcomes and satisfaction usually increase. This positive learning experience not only promotes students’ academic performance, but also facilitates their progress in innovation and problem-solving skills [23]. In summary, based on the perspective analysis of the conservation of resources theory, the usage of AI in teaching technology can effectively optimize the allocation of resources and task management, which in turn promotes the development of students’ innovative behaviors and abilities. By releasing cognitive resources, improving cognitive efficiency, and providing personalized support, teachers can create a more innovative learning environment for students in teaching.

AI can help teachers to automate some routine management and assessment tasks in teaching, such as homework correction and learning progress tracking. These tasks usually take up a lot of teachers’ time and energy. With the support of AI, teachers can reduce these loads, enabling them to focus more on interaction with students, personalized teaching, and care, and thus increase students’ well-being [24,25]. First, AI technologies can provide personalized learning paths and content recommendations, adapting teaching methods and materials to students’ learning styles and progress. This personalized learning experience can enhance students’ motivation and sense of achievement, thus promoting their well-being [26]. Second, when students receive more timely and accurate feedback through AI technology, their sense of learning achievement is usually enhanced. AI can help identify students’ learning weaknesses and provide targeted support, which can increase students’ self-confidence and satisfaction, thus enhancing their well-being [27]. Third, AI can provide additional social support and interaction opportunities through virtual assistants or online communities to help students build good academic and emotional relationships. Such social interactions are critical to students’ emotional health and well-being [28]. In summary, the usage of AI in teaching technology can help to reduce cognitive load, enhance the learning experience, increase the sense of achievement, and promote social interactions, and these factors together can significantly improve students’ well-being. By optimizing teaching and learning processes and personalizing support, teachers can create educational environments that are more conducive to students’ well-being and health.

Therefore, the following hypotheses are proposed in this study:

**H1:**  *The usage of AI in teaching significantly and positively affects students’ innovative behavior (a) and well-being (b)*.

### 2.3. The Mediating Role of Positive Emotion

AI technology can help teachers simplify and automate some tedious teaching tasks, such as homework correction and classroom management. In this way, teachers can focus more on the interaction with students and the provision of teaching content, which reduces the excessive intervention and negative impact on students, and thus promotes a more positive emotional experience for students [29,30]. Firstly, AI can provide personalized learning paths and support according to students’ learning progress and characteristics. When students feel that their learning needs are effectively met, they usually feel more accomplished and experience positive emotions because they are able to learn and make progress at their own pace [31]. Secondly, AI can provide immediate and personalized feedback to help students gain a clearer understanding of their learning progress and performance. This timely positive feedback can enhance students’ self-confidence and motivation and promote them to maintain a positive emotional state in learning [27]. Thirdly, when students receive more accurate learning support and feedback through AI technology, they tend to overcome learning problems and challenges more effectively. This successful experience and sense of achievement can promote students’ positive emotion and make them more willing to engage in learning [32]. In conclusion, the usage of AI in teaching technology can help to promote students’ positive emotion.

Positive emotional states contribute to the release and efficient use of cognitive resources. According to conservation of resources theory, individuals tend to conserve resources by reducing cognitive load in order to cope with complex tasks. When students are in a positive emotional state, their cognitive resources are more likely to be focused and effectively applied to innovative thinking and behaviors because positive emotion reduces feelings of stress and distraction, and enhances flexibility and creativity in thinking [33,34]. Firstly, positive emotion is usually accompanied by a positive attitude towards the learning task and higher levels of motivation. When students feel happy and fulfilled, they are more likely to be engaged in their learning and maintain a positive attitude toward challenging academic goals. This learning motivation and sense of achievement are important drivers of innovative behavior, as students tend to explore new ideas and solutions to maintain their academic success and personal satisfaction [27]. Secondly, positive emotions also promote good social interactions and support networks. Students are more willing to collaborate, share ideas, and receive feedback from peers and teachers in a positive emotional climate. This positive social environment helps innovative behavior to germinate and develop because students can be inspired and supported by diverse perspectives and experiences [34]. Thirdly, positive emotion helps to enhance students’ problem-solving ability and creative thinking. When students feel relaxed and happy, they are more able to adopt a flexible way of thinking and try new approaches and solutions. This kind of free thinking and creative exploration is the basis of innovative behavior, which helps students to show unique creativity and innovation in both academic and non-academic fields [20,31]. In summary, students’ positive emotions not only promote the performance of innovative behavior, but also enhances their well-being and mental health.

The use of AI technology can reduce teachers’ cognitive load spent on tedious administrative and assessment tasks, thus enabling teachers to focus more on the design and implementation of innovative teaching strategies [11]. The personalized support and feedback provided by teachers through AI in teaching can be more effective in promoting students’ motivation and sense of achievement, which in turn enhances students’ positive emotion. AI is able to provide customized learning paths and support based on students’ learning needs and characteristics [1]. When students feel that their learning is effectively supported by personalized support, they usually show higher motivation and positive emotions. This personalized learning experience not only helps students to progress academically, but also enhances their ability to solve problems and innovate [2]. The personalized support and immediate feedback that students receive through AI technology in a positive emotional state can help to release and effectively utilize their cognitive resources. Such released cognitive resources can motivate students to think and act more creatively as they are able to focus more on exploring new ideas and solutions rather than being distracted by tedious learning management tasks [7]. Positive emotional states and effective learning experiences supported by AI technology can enhance students’ well-being. Students typically experience higher levels of satisfaction and well-being when they feel that their learning is effectively supported and recognized. This well-being comes not only from academic achievement, but also from being able to enjoy the positive effects of personalized learning experiences and social interactions [15]. Therefore, the following hypothesis is proposed in this study:

**H2:**  *Positive emotion mediates between the usage of AI in teaching and innovative behavior*.

**H3:**  *Positive emotion mediates the relationship between teachers’ usage of AI in teaching and well-being*.

### 2.4. The Moderating Role of AI Trust

Students’ trust in AI can reduce cognitive load and emotional stress when interacting with AI [6]. Conservation of resources theory states that when individuals trust an external tool or system, they are more inclined to focus their cognitive resources on the task itself rather than on monitoring or having misgivings about the system. Therefore, if students have trust in the AI used in teaching and learning, they are able to engage in learning activities more easily and attentively when interacting with the AI, which helps to enhance their positive emotion [18]. Students’ trust in AI can enhance their acceptance and satisfaction with personalized learning experiences [35]. AI technologies are often able to provide customized learning support and feedback based on students’ learning needs and progress. If students trust this personalized AI support, they are more likely to feel understood and supported in the learning process, which enhances their motivation and positive emotion [36].

In addition, students’ trust in AI can make them more open to receiving feedback and support from AI. Effective feedback and support are important positive factors in the learning process and can help students better understand and cope with learning challenges [37]. When students trust AI to provide them with useful feedback, they are more likely to achieve positive psychological states in terms of academic achievement and self-efficacy, which further enhances their positive emotion. Therefore, students’ trust in AI can modulate the positive effects of teachers’ use of AI on students’ positive emotion, making the teaching process more productive and enjoyable, contributing to students’ academic success and psychological well-being [38]. Therefore, the following hypothesis is proposed in this study:

**H4:**  *AI trust moderates the effect of the usage of AI in teaching on positive emotion. Specifically, the stronger the AI trust, the stronger the positive effect of the usage of AI in teaching on positive emotion*.

Further, because positive emotion mediates the relationship between teachers’ usage of AI in teaching and innovative behavior and well-being, AI trust moderates the effect of teachers’ usage of AI in teaching on positive emotion. Therefore, the present study further suggests that AI trust can mediate the mediating role of positive emotion. Therefore, this study proposed the following hypothesis:

**H5:**  *AI trust moderates the mediating role of positive emotion in the process of the usage of AI in teaching’s influence on innovative behavior (a) and well-being (b)*.

**Figure 1:**
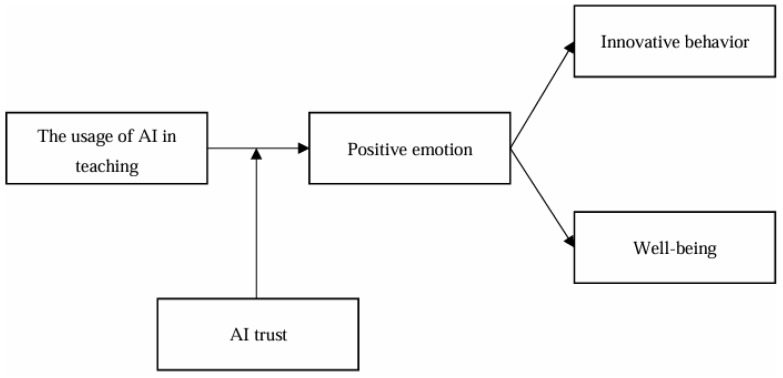
Research model.

In summary, the model diagram for this study is shown in Figure 1.

## 3. Method

### 3.1. Procedure and Sample

In this study, a questionnaire was used to collect data from university students in the Zhejiang Province of China. The researcher first imported the designed questionnaire into the questionnaire collection platform “Questionnaire Star” to generate the electronic link and QR code of the questionnaire, and then contacted the person in charge of the relevant university to assist in distributing the electronic link and QR code of the questionnaire. The questionnaires were filled out anonymously and the students’ wishes were fully respected. Since all variables in this study were self-assessed by the students, a two-stage questionnaire collection method was used in order to reduce the negative impact of common methodological bias on the data. In order to ensure the accurate matching of the questionnaire data, the subjects were asked to fill in the last 6 digits of their mobile phone numbers. The first round of research was conducted in May 2024, measuring information on the usage of AI in teaching, AI trust, and demographic variables. A total of 473 questionnaires were distributed and 432 were returned. After deleting unqualified questionnaires, such as those with too short response time and those with obvious patterns in the results, a total of 420 valid questionnaires were obtained, and the effective recovery rate of the questionnaires was 88.79%. The second round of the survey was conducted one month later, measuring positive emotion, innovative behavior, and well-being. This round mainly tracked the valid questionnaires from the first round, and 385 questionnaires were collected by matching the last 6 digits of the subjects’ mobile phone numbers. After deleting the qualified questionnaires, a total of 356 valid questionnaires were obtained, and the effective recovery rate of the questionnaires was 92.47%.

Among the valid samples, 62.08 per cent were male and 37.92 per cent were female; 33.21 per cent were aged 18–25; 25.14 per cent were aged 26–30; 35.21 per cent were aged 31–40; and 6.44 per cent were aged 41 and above.

### 3.2. Measure

All the variables in this study were selected from mature domestic and foreign scales, and the questionnaires for the foreign scales were designed in strict accordance with the procedure of translation-back-translation. To ensure the applicability of the translated foreign scales in the Chinese context, two doctoral students were invited to review the content of the scales. All questionnaires were measured on a 5-point Likert scale, where 1 stands for “totally disagree” and 5 stands for “totally agree”.

The usage of AI in teaching: The usage of AI in teaching was measured on a 3 item scale developed by Medcof [39]. The representative item was “I used AI to carry out most of my teaching functions”, and the internal consistency coefficient of this variable is 0.92.

Positive emotion: A 9 item scale developed by Watson et al. was selected to measure positive emotion [40]. The representative item was “happy”, and the internal consistency coefficient of this variable was 0.93.

AI trust: The 11 item scale developed by Chowdhury et al. was used to measure AI trust [41]. The representative item was “I feel trust in AI”, and the internal consistency coefficient for this variable was 0.87.

Innovative behavior: Innovative behavior was measured using a 9 item scale developed by Ng et. al. [42]. The representative item was “I create new ideas for improvements”. The internal consistency coefficient of this variable was 0.92.

Well-being: The 18 item scale developed by Zheng et al. was used to measure well-being [43]. Among them, the representative item was “My life is very interesting”. The internal consistency coefficient of this variable was 0.93.

Control variables: In this study, demographic variables such as age and gender were used as control variables. Age was a fill-in-the-blank question in which the subjects directly fill in their age, and gender is a multiple-choice question in which the subjects choose their gender according to their actual situation.

## 4. Results

### 4.1. Common Method Bias Test

We first tested for common method bias using the Harman one-way test. An exploratory factor analysis was conducted with the title terms of all variables unrotated, and the results showed that the eigenvalue of the first principal component was greater than 1 and explained 27.74% of the variance without exceeding the critical value of 40%, which indicated that the problem of common method bias in this study was not serious. Meanwhile, in view of the possible insensitivity of the results of the Harman one-way test method, this study added an error variable factor to the five-factor model. Subsequently, the model was compared with the five-factor model, and it was found that the changes in the indicators were not significant (CFI = 0.014, ΔTLI = 0.011, ΔRMSEA = 0.009), which again indicated that the common method bias problem in this study was not serious.

### 4.2. Descriptive Statistical Analysis

The mean, standard deviation of the variables involved in this study, and the correlation coefficients among the variables are shown in Table 1. As shown in Table 1, the usage of AI in teaching is significantly and positively correlated with positive emotion (r = 0.199, *p* < 0.001), innovative behavior (r = 0.295, *p* < 0.001), and well-being (r = 0.284, *p* < 0.001). Positive emotion was significantly and positively correlated with innovative behavior (r = 0.348, *p* < 0.001) and well-being (r = 0.364, *p* < 0.001). This tested the hypothesis of the present study to some extent.

### 4.3. Confirmatory Factor Analysis

In this study, validation factor analysis was conducted using Mplus 7.4. A five-factor model (benchmark model) was constructed, followed by a four-factor model, a three-factor model, a two-factor model, and a one-factor model, respectively, and the results are shown in Table 2. After comparing the factor models, it was found that the fitting indexes of the five-factor model (benchmark model) were significantly higher than those of other models (χ^2^ = 655.19, df = 220, χ^2^/df = 2.97, RMSEA = 0.07, CFI = 0.94, TLI = 0.93, SRMR = 0.05), indicating that the discriminant validity between the main variables is good and the research data passed the confirmatory factor analysis test.

### 4.4. Hypothesis Testing

**Figure 2:**
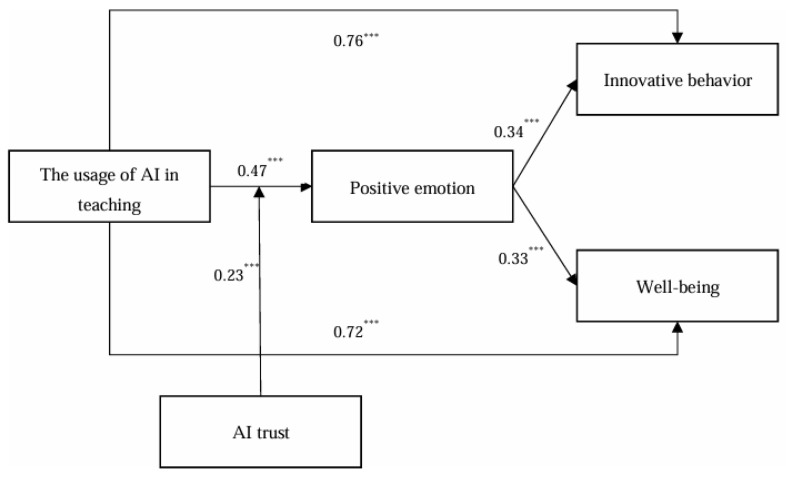
The result of the hypothesis test. (Note: *** *p* < 0.001).

In this study, the hypotheses were tested by constructing a structural equation model using Mplus 7.4. The coefficients and significance of the paths are shown in Figure 2.

1. Direct effect test

From Figure 2, it can be seen that the usage of AI in teaching significantly and positively affects students’ innovative behavior (B = 0.76, *p* < 0.001) and well-being (B = 0.72, *p* < 0.001). Therefore, H1 was validated. In addition, positive emotion significantly and positively influenced students’ innovative behavior (B = 0.34, *p* < 0.001) and well-being (B = 0.33, *p* < 0.001).

2. Test of mediating effect

In order to verify that positive emotion mediates the relationship between teachers’ usage of AI in teaching and innovative behavior and well-being, the mediating effect was tested in this study using the conditional indirect effects approach recommended by Preacher (Bootstrap = 5000). The results found that the mediating effect value of positive emotion between the usage of AI in teaching and innovative behavior was 0.15, and the confidence interval at the 95% level of the indirect effect was [0.13, 0.24], which did not contain 0. The mediating effect of positive emotion between the usage of AI in teaching and well-being was 0.16, and the confidence interval of the indirect effect was [0.08, 0.23], without 0, i.e., the mediating effect of positive emotion was established. Therefore, H2 and H3 were verified.

3. Moderating effect test

**Figure 3:**
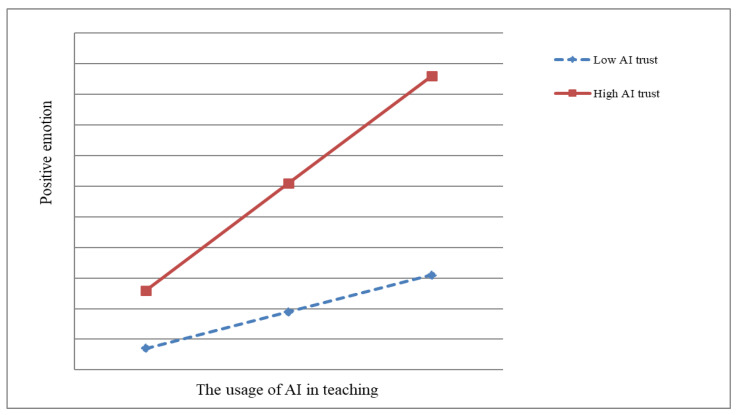
The moderate role of AI trust.

As can be seen in Figure 2, AI trust moderated the relationship between the usage of AI in teaching and positive emotion (interaction term coefficient B = 0.23, *p* < 0.001), and H4 was verified. In order to further test the hypothesis of H4, this study used the simple slope method to plot the moderating effect of AI trust between the usage of AI in teaching and positive emotion, as shown in Figure 3.

4. Test of mediated effect of moderation

This study used the Bootstrap method to test the mediated effect of being moderated by randomly sampling 5000 times, and the results are shown in Table 3. As shown in Table 3, when the trust degree in AI is low, the mediation effect value of positive emotion is 0.16, with 95% confidence interval [0.09, 0.25], not including 0. When the trust degree in AI is high, the mediation effect value of positive emotion is 0.05, with a 95% confidence interval [−0.10, 0.21], including 0. There is a significant difference between the two. Therefore, H5a is validated.

When AI trust is high, the mediation effect value of positive emotion is 0.03 with a 95% confidence interval [−0.34, 0.23], including 0. When AI trust is low, the mediation effect value of positive emotion is 0.15 with a 95% confidence interval [0.08, 0.36], including 0. There is a significant difference between the two. Therefore, H5b was validated.

## 5. Discussion

### 5.1. Theoretical Implication

Firstly, this study explores the mechanism in which the positive influence of AI technology changes students’ innovative behavior. This study reveals how the usage of AI in teaching technology significantly enhances students’ innovative behavior. By analyzing the mediating role of students’ well-being and positive emotion, the study highlights the potential of AI technology in stimulating students’ creativity and innovative potential. This finding helps to further understand the mechanism of AI technology in education and provides a new theoretical perspective for educational technology and innovation research.

Secondly, this study expands the research on AI trust by revealing the moderating effect of AI trust on teaching effectiveness. This study not only found the effects of students’ trust in AI technology on their positive emotion and well-being, but also showed how this trust moderated the effect of teachers’ usage of AI in teaching. This finding highlights the importance of emotional factors in the adoption of educational technology and provides a theoretical basis for future research to explore the introduction and application of AI technology in depth.

Thirdly, this study provides educators with practical strategies for integrating AI technologies to enhance teaching effectiveness and student well-being. However, while AI presents promising opportunities for innovation, its integration into education must be approached with caution. Over-reliance on AI risks reducing the teaching profession to mere facilitation and management roles. Educators should critically assess the long-term implications of AI on pedagogy, maintaining a balanced perspective that prioritizes ethical values, critical reflection, and the cultivation of human intelligence alongside artificial intelligence. By understanding how teachers can effectively use AI tools to enhance the learning experience and promote innovation, the study provides guidance on best practices for technology integration in educational practice. This has important practical implications for educational administrators and policy makers in promoting the development of modern educational technology.

### 5.2. Practical Implication

Firstly, teachers should integrate AI technologies to promote student innovation and well-being. Teachers can actively integrate AI technologies into their teaching, for example, by using smart educational tools, personalized learning software, or virtual assistants, which can provide a more personalized and interactive learning experience. However, particular caution should be exercised when using AI for evaluative tasks, such as grading or feedback generation. Evaluation is a core pedagogical activity with profound implications for learning, and over-reliance on AI in this area might risk standardization and reduce critical pedagogical engagement. Teachers should critically assess what to evaluate, why to evaluate, and how AI can support rather than replace evaluative judgment. Research has shown that the use of these technologies can significantly enhance students’ innovative behavior and well-being. Therefore, educators should explore and adopt AI tools that are appropriate for their content and student population in order to enhance their teaching effectiveness and learning experience.

Secondly, teachers should pay attention to students’ positive emotions and trust building. Students’ positive emotions play an important role in teaching and learning. At the same time, teachers need to address the growing over-reliance of students on AI technologies, which can undermine the development of critical thinking and natural intelligence. Building trust in AI among teachers is equally critical, as their skepticism may hinder effective integration. Transparent communication, professional development, and collaborative experimentation with AI tools can help teachers develop confidence in responsibly using AI while guiding students towards a balanced and ethical engagement. Teachers should not only pay attention to the delivery of teaching content, but also focus on creating a positive learning atmosphere and emotional support. Meanwhile, research has found that students’ trust in AI affects their positive emotions. Therefore, teachers need to build students’ trust in AI tools through transparent and effective communication when introducing and using AI technology. This sense of trust can help to enhance students’ interactive experience with AI technology, thus further promoting their positive emotions and learning effectiveness.

Thirdly, teachers should continuously evaluate and adjust the application strategies of AI technology. Educators should continually evaluate and adjust the use of AI technology to ensure its positive impact on students’ innovative behavior and well-being. This includes monitoring student feedback and performance to understand their acceptance and experience with AI tools. By collecting and analyzing data, teachers can adapt their teaching methods and the use of AI tools to maximize their educational impact and enhance the student learning experience.

### 5.3. Research Limitations and Future Research Perspectives

Firstly, the current study focuses on specific educational settings and the use of specific AI tools, thus the generalization of the results is limited. Future research can extend the study to different regions, education levels, and AI technology application scenarios to verify the consistency and generalizability of the results. For example, the use of AI technology in developing countries could be considered.

Secondly, the measurement tools of well-being and innovative behavior used in the study may be subjective and subject to measurement error, especially in cross-cultural and cross-linguistic studies. Future research could use multiple measures and multidimensional assessment tools to improve the credibility and comparability of the findings. Future research could explore and use multiple measures and multidimensional assessment instruments to improve the objectivity and comparability of well-being and innovative behavior measures. This includes validation in cross-cultural and cross-linguistic settings.

Thirdly, most research has focused on the short-term impact of AI technology, with limited understanding of long-term learning outcomes and sustainability. Future research could conduct long-term follow-up studies to explore the lasting effects of AI technology on student motivation, sustained innovative behavior, and long-term well-being. This requires long-term data collection and analysis to understand the long-term impact of AI technologies in education.

## Figures and Tables

**Table 1:** Mean, standard deviation, correlation, and reliability among study variables.

Variable	Mean	SD	1	2	3	4	5	6	7
1 Gender	1.379	0.486	1						
2 Age	20.225	3.643	0.011	1					
3 Well-being	3.634	1.584	0.097	0.076	1				
4 Innovative behavior	4.014	1.440	0.077	0.044	0.378 ***	1			
5 AI trust	2.531	0.757	0.016	0.128 *	0.064	0.018	1		
6 The usage of AI	4.360	1.491	0.084	0.016	0.284 ***	0.295 ***	0.161 *	1	
7 Positive emotion	3.090	1.179	−0.093	0.085	0.364 ***	0.348 ***	0.150 *	0.199 ***	1

Note: * *p* < 0.05, *** *p* < 0.001.

**Table 2:** Confirmatory factor analysis.

Model	χ^2^	df	χ^2^/df	CFI	TLI	RMSEA
Five-Factor Model	655.19	220	2.97	0.94	0.93	0.07
Four-Factor Model	1001.15	224	4.47	0.89	0.88	0.10
Three-Factor Model	1807.05	227	7.96	0.78	0.76	0.14
Two-Factor Model	2121.14	229	9.26	0.74	0.71	0.17
One-Factor Model	2818.43	230	12.25	0.64	0.61	0.18

**Table 3:** Moderated mediation effect test.

Dependent Variable	Moderator Variable	Indirect Effect	SE	95% CI	
Innovative behavior	Mean − 1 SD	0.16	0.04	0.09	0.25
	Mean + 1 SD	0.05	0.30	−0.10	0.21
Well-being	Mean − 1 SD	0.15	0.04	0.08	0.36
	Mean + 1 SD	0.03	0.45	−0.34	0.23

## Data Availability

The data that support the findings of this study are available from the first author upon reasonable request.
